# Deep Learning for Automated Detection of Cyst and Tumors of the Jaw in Panoramic Radiographs

**DOI:** 10.3390/jcm9061839

**Published:** 2020-06-12

**Authors:** Hyunwoo Yang, Eun Jo, Hyung Jun Kim, In-ho Cha, Young-Soo Jung, Woong Nam, Jun-Young Kim, Jin-Kyu Kim, Yoon Hyeon Kim, Tae Gyeong Oh, Sang-Sun Han, Hwiyoung Kim, Dongwook Kim

**Affiliations:** 1 Department of Oral & Maxillofacial Surgery, Yonsei University College of Dentistry, 50-1 Yonsei-ro, Seodaemun-gu, Seoul 03722, Korea; baachooo@yuhs.ac (H.Y.); evikle16@gmail.com (E.J.); KIMOMS@yuhs.ac (H.J.K.); CHA8764@yuhs.ac (I.-h.C.); YSJOMS@yuhs.ac (Y.-S.J.); OMSNAM@yuhs.ac (W.N.); JYOMFS@yuhs.ac (J.-Y.K.); SCVT8000@yuhs.ac (J.-K.K.); TONNYYHKIM@yuhs.ac (Y.H.K.); OTNK@yuhs.ac (T.G.O.); 2 Department of Oral & Maxillofacial Radiology, Yonsei University College of Dentistry, 50-1 Yonsei-ro, Seodaemun-gu, Seoul 03722, Korea; SSHAN@yuhs.ac; 3 Department of Radiology, Research Institute of Radiological Science, Yonsei University College of Medicine, 50-1 Yonsei-ro, Seodaemun-gu, Seoul 03722, Korea

**Keywords:** YOLO, deep learning, panoramic radiography, odontogenic cysts, odontogenic tumor, computer-assisted diagnosis, artificial intelligence

## Abstract

Patients with odontogenic cysts and tumors may have to undergo serious surgery unless the lesion is properly detected at the early stage. The purpose of this study is to evaluate the diagnostic performance of the real-time object detecting deep convolutional neural network You Only Look Once (YOLO) v2—a deep learning algorithm that can both detect and classify an object at the same time—on panoramic radiographs. In this study, 1602 lesions on panoramic radiographs taken from 2010 to 2019 at Yonsei University Dental Hospital were selected as a database. Images were classified and labeled into four categories: dentigerous cysts, odontogenic keratocyst, ameloblastoma, and no lesion. Comparative analysis among three groups (YOLO, oral and maxillofacial surgeons, and general practitioners) was done in terms of precision, recall, accuracy, and F1 score. While YOLO ranked highest among the three groups (precision = 0.707, recall = 0.680), the performance differences between the machine and clinicians were statistically insignificant. The results of this study indicate the usefulness of auto-detecting convolutional networks in certain pathology detection and thus morbidity prevention in the field of oral and maxillofacial surgery.

## 1. Introduction

The cysts and tumors of the jawbone are usually painless and asymptomatic unless they grow so large as to involve the entire jawbone, causing noticeable swelling or weakening it to cause pathologic fractures [1,2]. Such late-stage radical surgery, involving ablation and reconstruction accompanying bone grafts and free flaps, drastically affects patients’ lives, causing facial deformity and subsequent social and emotional incompetence [3,4]. Although rare, a carcinomatous change of benign jaw lesions has also been described in the literature [5,6]. The asymptomatic nature of such lesions in the initial stage leads to delayed diagnosis and subsequent poor treatment outcome [7]. Early diagnosis is the only option to ensure healthy years of life [8,9].

The majority of these lesions can be identified at an earlier stage in dental clinics through a routine radiographic exam called panoramic radiograph, or orthopantomogram [10]. In fact, cystic lesions are often identified as incidental findings on panoramic radiographs, with no apparent symptoms regardless of the patient’s chief complaint [8]. However, accurate interpretation requires training and can be challenging even for experienced professionals, which is mainly due to the process of panoramic radiography itself, whereby the image is captured by a sensor/plate that rotates around the patient’s head, causing superimposition of all the bony structures of the facial skeleton [11,12].

Deep convolutional neural networks (DCNNs) are gaining increased attention in the field of medical imaging. A deep learning tool for image detection, YOLO, is characterized by its simple data processing network, which can both detect and classify an object at the same time, while also providing faster image analysis than Faster Region-based convolutional neural networks (Faster-RCNN) [13]. We hypothesized that with adequate training data, YOLO would show decent performance as a computer-assisted diagnosis (CAD) system. Moreover, it would support clinicians in formulating second opinions or reconfirming the detection and diagnoses of odontogenic cysts and tumors that appear on the panoramic radiograph.

Along with new technologies to study the maxillofacial region, several studies on the automatic detection of odontogenic cysts and tumors have been published [14,15,16,17,18]. However, to our knowledge, this study utilizes the largest dataset for automatic detection targeting maxillofacial lesions, and it is the first study comprising both maxilla and mandible datasets.

This study includes comparative analysis among three groups: YOLO, oral and maxillofacial surgery (OMS) specialists, and general practitioners (GP). Detecting and classifying performance was measured in multiple ways in order to evaluate the suitability of YOLO as a CAD system.

## 2. Materials and Methods

### 2.1. Patients Selection and Data Collection

**Figure 1:**
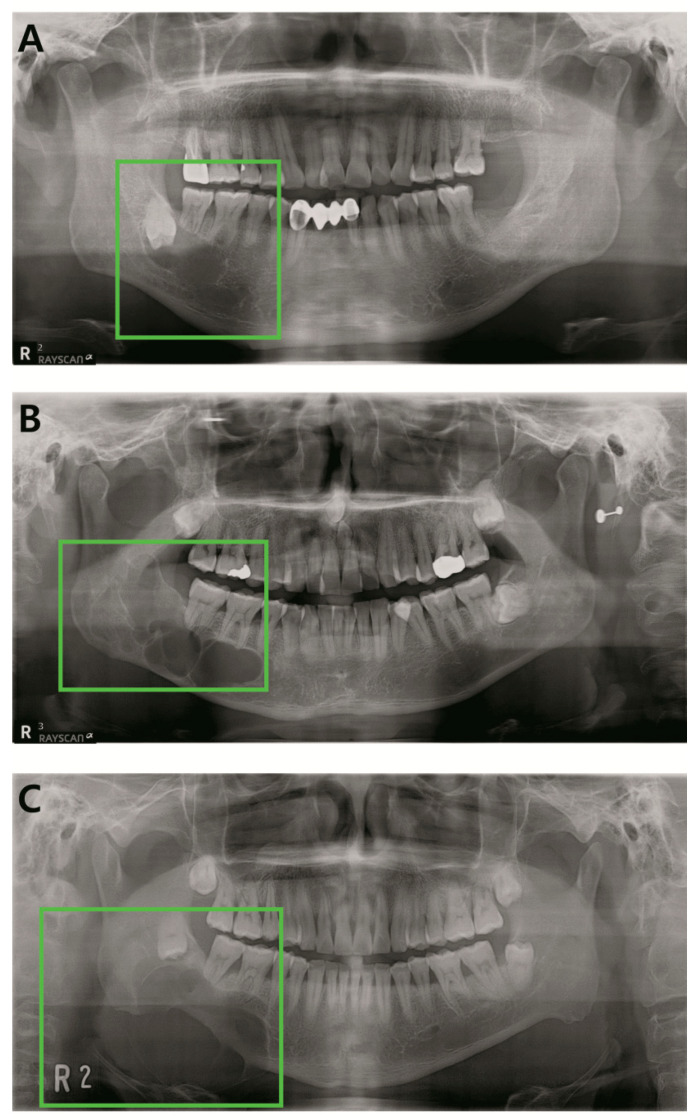
Examples of the included lesions. (**A**) Dentigerous cyst, (**B**) odontogenic keratocyst (OKC), (**C**) ameloblastoma.

Panoramic radiographs of histopathologically confirmed cyst and tumors of the jawbone were included for this study. Dentigerous cyst, odontogenic keratocyst (OKC), and ameloblastoma were the included diagnoses (Figure 1). Only the preoperative radiographs were included, postoperative radiographs being excluded. A total of 1603 panoramic radiographs taken from 2010 to 2019 at Yonsei University Dental Hospital were obtained (Table 1). Demographic data of the study subjects (N = 1603).

The digital panoramic radiographs of all patients were obtained in the Department of Oral and Maxillofacial Radiology, Yonsei University Dental Hospital.

This study was approved by the Institutional Review Board (IRB) of Yonsei University Dental Hospital (Approval number: 2-2018-0062).

### 2.2. Annotation of Images

Ground truth panoramic images were labeled with the YOLO mark according to previously confirmed histopathologic diagnosis. The images were labeled into four categories: dentigerous cysts, odontogenic keratocyst, ameloblastoma, and no lesion.

### 2.3. Pre-Processing and Image Augmentation

Datasets were randomly split into two mutually exclusive sets, training and testing (Table 1. Demographic data of the study subjects (N = 1603). To minimize overfitting issues that may arise when a small dataset is utilized for deep learning, we augmented our training set by applying transformation methods. Images were horizontally and vertically flipped (in the range of 10°), translated, and scaled, obtaining 16,224 augmented training set [ {1422−174 (validation set)} × 13] and 181 testing set. This work was conducted with the Pytorch 1.2.0 framework with Python 3.7.4 on a GPU of NVIDIA Quadro P5000.

### 2.4. YOLO Architecture and Workflow

YOLO starts with dividing an input image (panoramic radiograph) into S × S non-overlapped grid cells. Each grid cell is responsible for detecting the potential lesion belonging to that cell. Furthermore, each grid cell consists of 2 bounding boxes, and bounding boxes are assigned confidence scores.

The class-specific confidence score for each class can be calculated as follows:

Pr (Class i | lesion ) × Confidence = Pr (Class i) × IOU ground truth predicted

**Figure 2:**
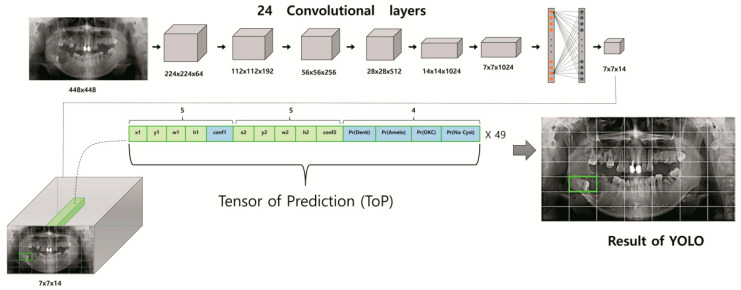
Schematic diagram of You Only Look Once (YOLO)-based computer-assisted diagnosis (CAD) structure.

YOLO’s single workflow pipeline consists of 24 convolutional layers with different kernel sizes, max-pooling layers with a size of 2 × 2, activation functions, and two fully connected layers [19]. At the end of the process, the tensor of prediction (ToP) with size of S × S × (5 × M + C) is generated, where S × S, M, and C are the number of grid cells, bounding boxes, and classes (i.e., odontogenic keratocyst, dentigerous cyst, ameloblastoma, and no lesion), respectively. Unknowns of ToP formulas are selected as follows. Since our study dealt with four classes of odontogenic lesion (i.e., odontogenic keratocyst, dentigerous cyst, ameloblastoma, no lesion), we set C = 4. Each grid cell, the smallest unit responsible for detection and classification, can be assigned various numbers, but in this study, a size of 7 × 7 (i.e., S = 7) was chosen for optimum efficiency. To get the best predicted box among the inner and outer boundaries of the object in the panoramic radiograph, we selected M = 2. The bounding box with the highest confidence score was automatically selected as the predicted box. The final output of the YOLO network represents a 3D matrix of ToP (tensor of prediction) with a size of 7 × 7 × 14. Each grid cell of the panoramic radiograph is expressed by 14 elements in the tensor. The first five elements correspond to the predictions of the first bounding box, while the second five elements are for the second bounding box. For each box, these elements represent the prediction information of the mass locations x, y, w, h, and confidence probability. The (x, y) coordinates correspond to the center of the box within the bounds of the grid cell. The width and height (w, h) are assigned in relation to the entire image. Finally, the confidence prediction represents the intersection over union (IOU) between the predicted box and any ground truth box. The last four elements (i.e., Pr OKC, Pr ameloblastoma, Pr dentigerous cysts, Pr no lesion) in the ToP represent the confidence scores of the class probabilities for each class. The bounding box with the highest confidence score (i.e., the highest IOU with ground truth) is selected. Since YOLO predicts only one bounding box for each grid cell responsible for detecting the mass location and assigning its appropriate class, the remaining bounding box is discarded. Additionally, among all the potential predicted lesions in each panoramic radiograph, YOLO only selects those boxes with confidence scores greater than a particular threshold. The Darknet framework is utilized for all training and testing processes. The overall schematic diagram of the YOLO-based CAD structure is presented in Figure 2. YOLO v2 was the implemented architecture model.

### 2.5. Performance Evaluation Method

YOLO’s diagnostic performance was evaluated in multiple ways. The average time spent analyzing the test set was measured among the three groups (YOLO, OMS specialists, GP) in terms of descriptive analysis (mean ±SD). Precision (1), recall (2), accuracy (3), and F1 score (4) were used as indicators for object detection assessment and classification performance. To quantitatively visualize the classification capability of YOLO, confusion matrices for the three groups were designed, and accuracy and F1 scores calculated. Furthermore, precision and recall among the three groups were statistically analyzed on a Kruskal–Wallis test with a statistical significance of p < 0.05. Statistical analyses were performed using R (Version 3.6.1, R Project for Statistical Computing, Vienna, Austria).

(1) $$\mathrm{Precision}=\frac{\mathrm{TP}\;}{\mathrm{TP}+\mathrm{FP}}$$

(2) $$\mathrm{Recall}=\frac{\mathrm{TP}\;}{\mathrm{TP}+\mathrm{FN}}$$

(3) $$\mathrm{Accuracy}=\frac{\mathrm{TP}+\mathrm{TN}}{\mathrm{TP}+\mathrm{TN}+\mathrm{FP}+\mathrm{FN}}\;$$

(4) $$F1\;\mathrm{score}\;=\frac{2\;\times \;(\mathrm{Recall}\;\times \;\mathrm{Precision})}{\mathrm{Recall}+\mathrm{Precision}}$$

TP: true positive, FP: false positive, FN: false negative, TN: true negative

## 3. Results

**Figure 3:**
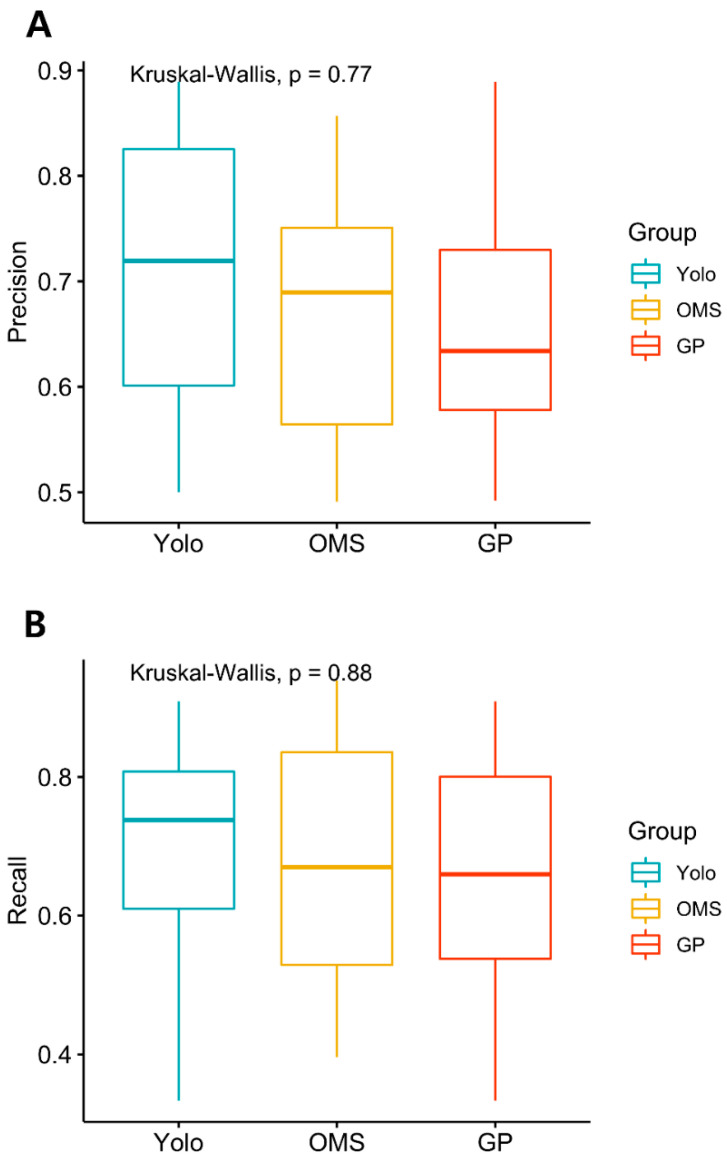
(**A**) Kruskal–Wallis test was performed to analyze the statistical significance of precision and recall among three groups (YOLO, OMS specialists, GP) A. Precision (**B**). Recall (p < 0.05). OMS: Oral and maxillofacial Surgery, GP: General practitioner.

**Figure 4:**
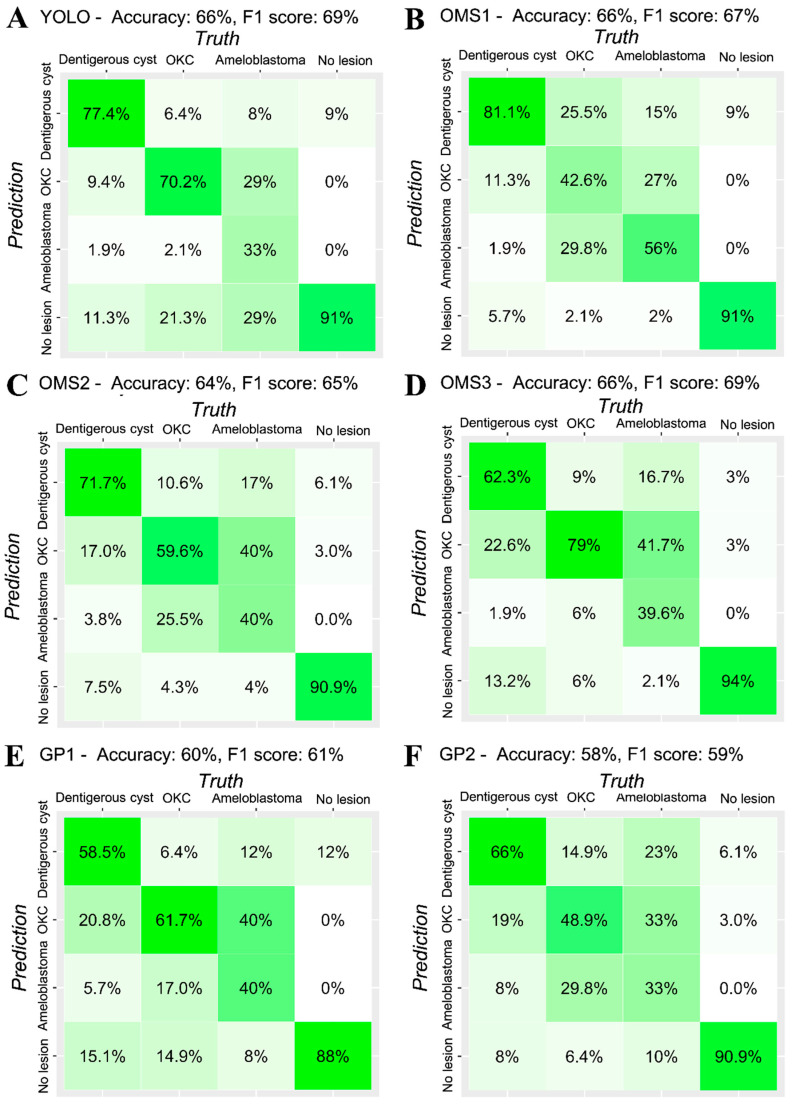
Confusion matrix of (**A**)- YOLO, (**B**), (**C**), (**D**)- OMS specialists (OMS), and (**E**), (**F**)- general practitioner (GP).

The average time to evaluate all the images of the test datasets (181) for human clinicians (OMS surgeons and GPs) was 33.8 (SD = 5.3) minutes, while YOLO showed real-time detection and classification performance. While the precision and recall of YOLO scored highest among the three groups, the difference was statistically insignificant, implying indistinguishable diagnostic performance (Table 2, Figure 3). Figure 4 presents the confusion matrix, showing the diagnostic outcomes of each class. The diagnostic accuracy of YOLO resulted in 0.663 while OMS2 and GP1 showed slightly lower accuracy (0.635 and 0.597), OMS3 ranked highest, with a F1 score of 0.694, followed by YOLO (0.693), GP2 (0.693), OMS1 (0.673), OMS2 (0.649), and GP1.

## 4. Discussion

Until recently, no artificial system or device could replace the human cognitive system, which is fast, accurate, and flexible [19]. Medical imaging in particular was considered an inviolable field requiring expert analysis and confirmation. However, remarkable developments in deep learning models, particularly the deep convolutional neural network (CNN) architecture, has yielded remarkable results exceeding those of human experts [20,21]. Through this research, we experienced the benefits and limitations of the auto-detecting deep CNN algorithm YOLO in detection and diagnosis on panoramic images of odontogenic cysts and tumors including dentigerous cysts, OKCs, and ameloblastoma. We have confirmed the feasibility of its use in clinical practice as a CAD system.

YOLO has some outstanding features that many systems lack. Unlike other classifier-based methods, YOLO is a real-time detection system that detects and classifies targeted objects simultaneously within a single network. RCNNs, widely adopted in many deep learning studies, use region proposal methods to first generate potential bounding boxes in an image and then run a classifier on these proposed boxes [19,22,23,24]. After classification, post-processing is used to refine the bounding boxes, eliminate duplicate detections, and rescore the boxes based on other objects in the scene. These complex pipelines are slow and hard to optimize, because each individual component must be trained separately. On the other hand, YOLO frames detection as a regression problem that does not require a complex pipeline nor semantic segmentation, which is burdensome but mandatory for most deep learning detection systems [19,25]. The region of interest (ROI) does not need manual framing because YOLO offers a powerful functionality in that it can learn ROIs and their background at the same time. YOLO’s neural network is simply activated on a new image at test time to predict detections. The YOLO base network runs at 45 frames per second with no batch processing on a Titan X GPU, and a fast version runs at more than 150 fps. YOLO can process streaming video in real time with less than 25 milliseconds of latency. In this study, YOLO actually performed detection and classification of the entire test set almost virtually instantaneously. Meanwhile, the average time for clinicians including board-certified specialists to evaluate all the images of the test datasets was 33.8 min (SD = 5.3). Considering that the pathology-locating ability of YOLO was statistically equivalent to that of the clinicians involved in our study, such detection speed constitutes a definitive advantage in designing CAD.

Fine-tuning the model is essential to optimizing the learning performance. Critical components determining the model learning performance are the control of overfitting and learning rate. YOLO slowly raises the learning rate and adjusts the number of epochs to reach the optimum stage. Furthermore, to avoid overfitting, the system uses dropout and extensive data augmentation. A dropout layer with a rate = 5 after the first connected layer prevents co-adaptation between layers [26]. For data augmentation, horizontal and vertical flipping (in the range of 10°), translating, and scaling are applied in the training phase [19].

Moreover, YOLO has high contextual understanding of the image, similar to that of the human cognitive system. It endeavors to analyze the whole image to predict each bounding box and predicts all bounding boxes across all classes for an image simultaneously. This significantly lowers the false positive rate (background errors). In fact, in the present study, YOLO’s false positive error was similar to and even lower than that of human clinicians.

**Figure 5:**
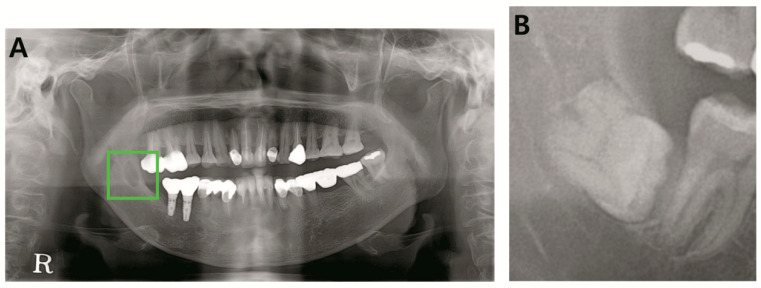
Misclassified early pathology (**A**). Early stage ameloblastoma (**B**). Early stage dentigerous cyst.

Odontogenic tumors and cysts do not reveal their distinct radiological characteristics until they reach a certain size. Early radiological appearances of odontogenic cysts and tumors are so indistinguishable from each other that even experienced oral and maxillofacial specialists are unable to guarantee their classification (Figure 5). Unfortunately, they are also asymptomatic during their progressive stage [27,28]. Due to such features of odontogenic cysts and tumor, relatively prevalent cysts such as dentigerous cysts and odontogenic keratocysts may turn out to be a threat to patient life quality if they are oversized or cause subsequent pathologic fracture [29,30]. Many types of ameloblastomas are more destructive in their progressive aspect. The infiltrative pathology frequently requires wide excision, which is often followed by simultaneous reconstruction, including free-flaps [31,32]. It cannot be denied that the detection and classification of pathology are both crucial components of an automatic diagnostic system. However, in assessing deep learning systems targeting odontogenic cysts and tumors, detection is more urgent in early stages, when radiological features are ambiguous. In fact, YOLO scored the highest detection rate, as represented by recall, and its consistency was confirmed by highest precision, although statistically insignificant. However, precision and recall are indicators that may vary according to the model’s threshold value. Thus, in order to quantify YOLO’s performance in balance, the F1 score was calculated, and only one OMS surgeon outranked YOLO. While the classification accuracy of the system did not outperform specialists, the results of this comparative analysis suggest the system’s potential as a powerful tool for computer-aided detection.

To our knowledge, this study comprises the largest number of panoramic radiographs to date among published deep learning studies on the detection of maxillo-facial cysts and tumors [14,15,16]. Unlike mandible lesions, radiological images of maxillary lesions are indistinct due to the overlay of anatomic structures such as maxillary sinus. However, we included cysts and tumors of both maxilla and mandible for training in order to eliminate selection bias. Ariji et al. published a deep learning study using 210 images of radiolucent lesions of mandibles [14]. Wiwiek’s study comprises 500 images, focusing on only two pathologies [15].

Panoramic radiography, the most widely used diagnostic tool for dentists, visualizes the entire maxillo-mandibular region on a single film. In addition to the dento-alveolar areas, the maxillary region, extending to the superior third of the orbits, and the entire mandible, extending as far as the temporomandibular joint region, are also included in the examination. Panoramic radiographs are especially beneficial in detecting odontogenic cysts and tumors, which almost without exception appear in the maxilla-mandibular lesion [33]. Meanwhile, in many countries, panoramic radiographs are not included in national health checkup programs. For example, in South Korea, a periodic health checkup includes an interview examination and posture test, a chest X-ray, a blood test, a urine test, and an oral examination. The oral examination exclusively relies on a visual inspection and formal questionnaires [34]. If panoramic radiography was to be utilized as a screening tool in combination with an auto-detecting system such as YOLO, clinicians with less experience in OMS or other specialty physicians such as general practitioners, endodontists, or periodontists would certainly achieve the early detection of maxilla-mandibular pathology on a much larger scale than is presently possible. YOLO could be useful for oral and maxillofacial specialists in generating preliminary opinions and in double-checking diagnoses, especially in cases of early stage odontogenic cysts and tumors that could have been missed due to insufficient experience, low clinical suspicion, or simple misdetection. A combination of YOLO’s diagnostic performance and systematic consultation to oral and maxillofacial specialists would dramatically decrease the rate of ablative surgery due to odontogenic cysts and tumors.

**Figure 6:**
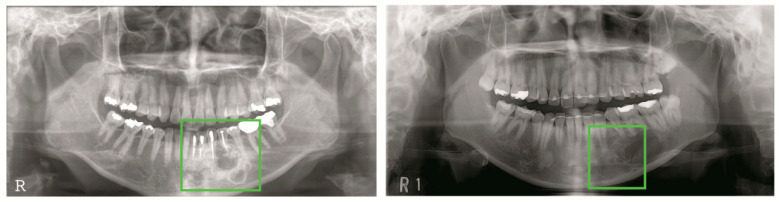
Small objects concentrated within a region of interest (ROI).

**Figure 7:**
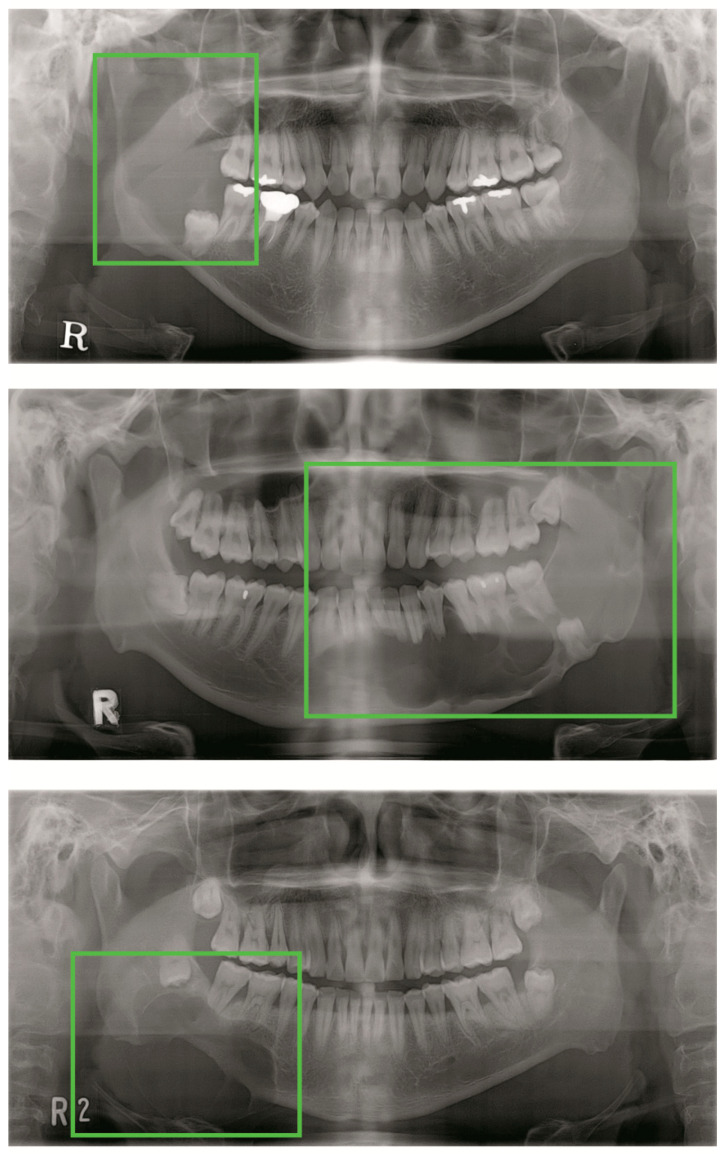
Undetected large lesions.

Despite the many benefits of YOLO mentioned above, this deep learning CNN model, as in any other architecture, is not omnipotent. Occasionally, YOLO struggles to localize objects correctly. Localization errors account for more of YOLO’s errors than all other sources combined. Specifically, each grid cell is accompanied by two bounding boxes and one final class, which confuses YOLO when small objects cluster within themselves (Figure 6). Odontogenic cysts and tumors appear in panoramic radiograph with various features and borders. Thus, YOLO might have a hard time when applied to pathologies with untrained aspect ratios or configurations. Moreover, feature maps that have inevitably passed through multiple convolutional and max-pool layers might have become too obscure to set the bounding boxes. Finally, large pathologies with large, hollow cores will present a large area of radiolucency on a panoramic radiograph. As a result, the larger the pathology, the higher the probability that multiple grid cells will recognize it as an absent lesion (Figure 7). These considerations may have contributed to the relatively significant false negative rate of YOLO in this study.

**Figure 8:**
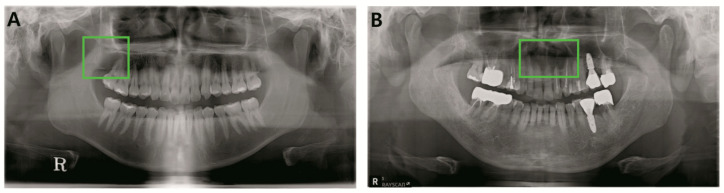
Undetected lesions for both YOLO and clinicians. (**A**). OKC, maxilla right. (**B**). OKC maxilla anterior.

**Figure 9:**
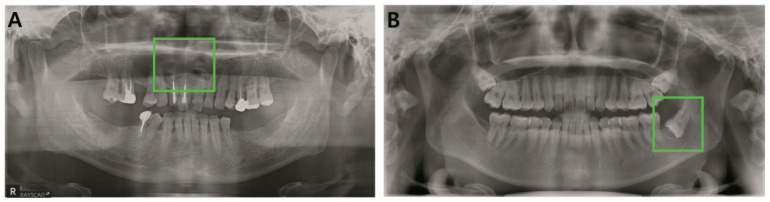
(**A**). Maxilla OKC was correctly detected and classified by YOLO while one-third of clinicians failed detection. (**B**). Mandible dentigerous cyst was correctly diagnosed by YOLO while two-thirds of clinicians failed detection.

However, YOLO cannot be solely blamed for the false negative rate in this study, which included radiologically ambiguous early stage pathologies and lesions of maxilla that even experienced clinicians have trouble definitively diagnosing. As mentioned, some lesions of maxilla are obscured by low bone density and the many adjacent anatomic structures which intersect with the target on the panoramic radiograph. Odontogenic keratocysts on maxilla were undetected by both YOLO and two-thirds of clinicians, including specialists and general practitioners (Figure 8). However, surprisingly, there were several occasions when YOLO detected and correctly classified lesions that clinicians had failed to recognize (Figure 9).

Moreover, as Gulshan et al. emphasized, in order for DCNN to achieve maximum performance, a few essential pre-requisites must be met [21]. Most importantly, there must be a large developmental set with tens of thousands of abnormal cases. In Gulshan’s study, performance on the tuning set saturated at 60,000 images; however, he suggested that additional gains might be obtained by increasing the diversity of training data (i.e., data from various clinics) [21]. Cha et al. also showed significantly varying accuracy by increasing the number of training datasets [35].

The prevalence of odontogenic cysts and tumors varies from 3.45% to approximately 33.8% according to geographic area. Radicular and dentigerous cysts comprise 70–90% of prevalent lesions while other pathologies occur relatively rarely [36]. This unbalanced distribution of odontogenic cysts and tumors poses a major obstacle to obtaining balanced medical data within a single institutional study. Despite utilizing the largest number of training data among similar studies and the data augmentation in training, the training dataset may have been unsatisfactory in terms of absolute size for optimum YOLO performance.

Further studies may be required to maximize the YOLO performance. First, combining two or more convolutional networks may have a synergistic effect on the general performance. For example, fast R-CNN yields far fewer localization errors but far more background errors, which can result in a high false positive rate, while the converse is true for YOLO. The Pascal Visual Object Classes (VOC) challenge includes a collection of datasets for object detection. It provides standardized image data sets for object class recognition, which enables an evaluation and comparison of different artificial network architectures. During the 2012 VOC competition, a combination of YOLO and Fast R-CNN significantly raised the mean accuracy precision outscoring solo performances of YOLO and Fast R-CNN. Several studies have combined different models to improve the classification accuracy [37,38]. However, there is a caveat in extracting feature sets from multiple models, due to potentially redundant information as the number of parameters increases. Secondly, in our study, we did not provide YOLO with external patient factors other than panoramic images. However, odontogenic cysts and tumors are characterized by their prevalence related to factors such as anatomical location, age group, sex, and ethnic background. Training the machine classifier with both image and non-image information may result in a better diagnosis rate. Lastly, adjusting the number of grid cells and bounding boxes might increase YOLO’s performance. As mentioned above, large pathologies and multiple small clustered lesions were occasionally undetected. In this study, for optimum performance, we set the number of grid cells and bounding boxes to 49 and 2, respectively. However, the purpose-driven setting of grid cell numbers and bounding boxes may yield better results in different circumstances.

In conclusion, within the limitations of this study, a real-time detecting CNN YOLO trained on a limited amount of labeled panoramic images showed diagnostic performance at least similar to that of experienced dentists in detecting odontogenic cysts and tumors. A range of factors that affected performance should be carefully considered in future studies. The application of CNNs in dental imagery diagnostics seems promising for assisting dentists.

## Figures and Tables

**Table 1:** Demographic data of the study subjects (N = 1603).

Characteristics	Training Set	Testing Set
	(N = 1422)	(N = 181)
**Age** [IQR]	42.0 [31.0; 53.0]	37.0 [25.0; 48.0]
**Diagnosis**		
Dentigerous cyst	1042 (73.3%)	52 (28.7%)
OKC	268 (18.8%)	48 (26.5%)
Ameloblastoma	112 (7.9%)	48 (26.5%)
No lesion*	0 (0.0%)	33 (18.2%)
**Sex**		
Female	455 (32.0%)	62 (34.3%)
Male	967 (68.0%)	119 (65.7%)
**Location**		
Mandible	1246 (87.6%)	125 (69.1%)
Maxilla	176 (12.4%)	23 (12.7%)
No lesion*	0 (0.0%)	33 (18.2%)

IQR: Interquartile range, OKC: Odontogenic keratocyst; *Panoramic radiograph without pathologic lesion was only used for testing.

**Table 2:** Precision and recall of YOLO, OMS specialists, and general practitioner (GP).

**Precision**	**Dentigerous Cyst**	**OKC**	**Ameloblastoma**	No Lesion*	**Mean (sd)**
**YOLO**	0.804	0.635	0.889	0.500	0.707 (0.174)
**OMS specialist**					0.671 (0.124)
OMS1	0.662	0.513	0.643	0.857	0.669 (0.142)
OMS2	0.717	0.491	0.576	0.789	0.643 (0.135)
OMS3	0.717	0.529	0.826	0.738	0.703 (0.125)
**GP**					0.658 (0.138)
GP1	0.705	0.492	0.633	0.604	0.608 (0.089)
GP2	0.804	0.635	0.889	0.500	0.707 (0.174)
**Recall**	**Dentigerous Cyst**	**OKC**	**Ameloblastoma**	No Lesion*	**Mean (sd)**
**YOLO**	0.774	0.702	0.333	0.909	0.680 (0.246)
**OMS specialist**					0.673 (0.203)
OMS1	0.811	0.426	0.563	0.909	0.677 (0.222)
OMS2	0.717	0.596	0.396	0.909	0.654 (0.215)
OMS3	0.623	0.787	0.396	0.939	0.686 (0.233)
**GP**					0.649 (0.21)
GP1	0.585	0.617	0.396	0.879	0.619 (0.199)
GP2	0.774	0.702	0.333	0.909	0.68 (0.246)

*No lesion: Panoramic radiograph without pathologic lesion; OMS: Oral and maxillofacial surgery, GP: General practitioner.
